# Organized Toe Maps in Extreme Foot Users

**DOI:** 10.1016/j.celrep.2019.08.027

**Published:** 2019-09-10

**Authors:** Harriet Dempsey-Jones, Daan B. Wesselink, Jason Friedman, Tamar R. Makin

**Affiliations:** 1 Institute of Cognitive Neuroscience, University College London, London WC1N 3AZ, UK; 2 Wellcome Centre for Integrative Neuroimaging, University of Oxford, Oxford OX3 9DU, UK; 3 Physical Therapy Department, Sackler Faculty of Medicine, Tel Aviv University, Tel Aviv 699 7801, Israel; 4 Sagol School of Neuroscience, Tel Aviv University, Tel Aviv 699 7801, Israel

**Keywords:** adaptive, amputees, deprivation, ecological behavior, fMRI, hand, plasticity, reorganization, sensorimotor, somatotopy

## Abstract

Although the fine-grained features of topographic maps in the somatosensory cortex can be shaped by everyday experience, it is unknown whether behavior can support the expression of somatotopic maps where they do not typically occur. Unlike the fingers, represented in all primates, individuated toe maps have only been found in non-human primates. Using 1-mm resolution fMRI, we identify organized toe maps in two individuals born without either upper limb who use their feet to substitute missing hand function and even support their profession as foot artists. We demonstrate that the ordering and structure of the artists’ toe representation mimics typical hand representation. We further reveal “hand-like” features of activity patterns, not only in the foot area but also similarly in the missing hand area. We suggest humans may have an innate capacity for forming additional topographic maps that can be expressed with appropriate experience.

## Highlights

• We ask if extreme behavior can cause the (re)emergence of somatotopic maps
• We investigated two foot artists, born without arms
• 7T fMRI shows individuated maps of up to 5 toes in the artists but not controls
• Activity in artists’ foot and hand areas was more “hand-like” than in controls

## Graphical Abstract

**Figure:**
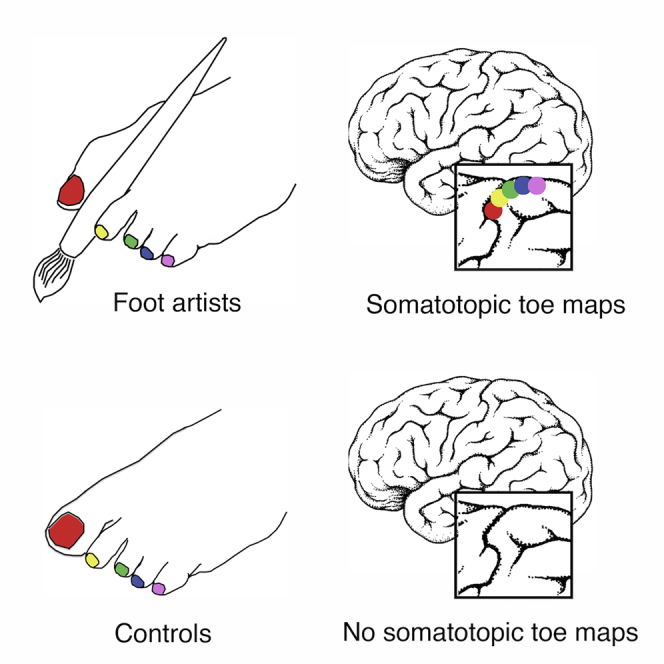

## Teaser

Dempsey-Jones et al. investigate two foot artists, born without arms, asking if a lifetime of radically altered bodily experience can cause new body maps in the brain. We provide evidence that complex toe behavior supports organized “hand-like” activity in the artists’ foot and (missing) hand areas, significantly more so than in controls.

## Introduction

The hand area of the primary somatosensory cortex (SI), and Brodmann area (BA) 3b in particular, contains detailed digit maps, with physically adjacent digits showing greater representational overlap than non-adjacent fingers (Besle et al., 2014, Kaas et al., 1979, Sanchez-Panchuelo et al., 2012, Thakur et al., 2012) (see Figures 2A and 3C). Although the gross features of the canonical hand representation are highly consistent in humans (Ejaz et al., 2015, Kikkert et al., 2016, Kolasinski et al., 2016), the inter-finger selectivity may be shaped by experience, e.g., digits used more frequently together in daily life show more representational overlap (Ejaz et al., 2015) and vice versa (Gindrat et al., 2015).

**Figure 1:**
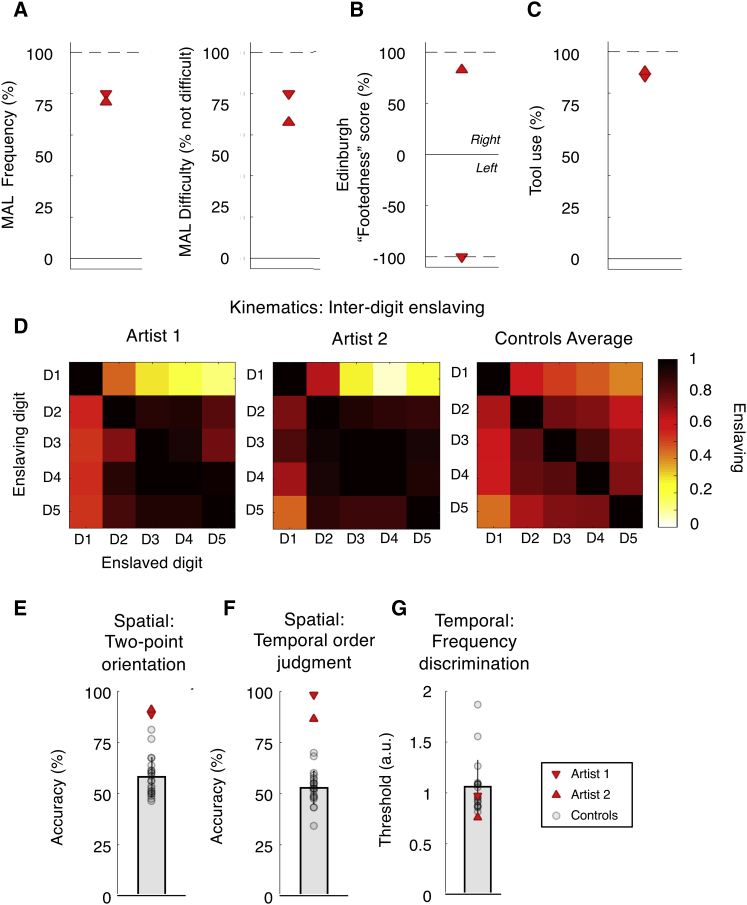
Assessment of Hand-like Foot Use in the Artist (A–C) Clinical (motor activity log) (A), standard (handedness) (B), and expert (tool use) (C) questionnaire scores show close-to-ceiling appropriation of the feet for daily life functions normally involving hands. Dashed lines indicate theoretical maxima. (D) Kinematic toe individuation in free-movement across digit pairs. Artists’ mean kinematics were not different from controls (n = 9). (E) Temporal order judgement (on D1). (F) Two-point orientation discrimination (on D1–D3). (G) Temporal frequency discrimination (on D1). Both artists displayed superior inter- and intra-digit tactile spatial acuity compared to controls but not temporal tactile acuity. Error bars show SD across controls. See also Table S1 and Video S1.

We investigated whether extreme habitual foot behavior might associate with organized toe maps in SI, where they have not been found in typically developed humans (Akselrod et al., 2017), although they are identified in monkeys (Liao et al., 2016, Nelson et al., 1980). Using 7 tesla neuroimaging during passive toe touch, we studied the foot representation of two individuals with developmental upper-limb amelia. Displaying exquisite compensatory adaptation, these individuals perform typically manual daily living tasks (e.g., dressing, feeding, and typing) with their feet (see Figures 1A–1C). Both use one foot for dextrous object manipulation and the other for stabilizing. Both are sufficiently skilled with their dextrous foot to allow writing, drawing, and painting to a level that supports their profession as artists (two of only three such foot artists in the UK).

We studied toe activity patterns in the artists’ SI foot area and sensorimotor (missing) hand area. Previous studies indicate that uni- or bilateral congenital hand absence leads to remapping of inputs from the mouth and feet into the deprived hand territory (Hahamy et al., 2017, Hahamy and Makin, 2019, Stoeckel et al., 2009, Striem-Amit et al., 2018, Yu et al., 2014, Yu et al., 2006). However, it is unclear whether such remapping truly represents recruitment of this zone to support toe function or aberrant activity, e.g., due to deprivation-driven inhibition reduction (Hahamy et al., 2017). To step beyond this highly common traditional approach of remapping in deprived cortex, we asked whether the missing hand cortex contained functional representational features of hand representation. Specifically, we focused on two critical representational hallmarks of hand representation in the primary sensorimotor cortices (BA 1–4), i.e., inter-digit selectivity and overlap patterns (Ejaz et al., 2015, Kikkert et al., 2016).

We anticipated that daily foot use would replace hand functions to drive rich and complex sensorimotor input patterns (Dempsey-Jones et al., 2016), which will further interplay with cognitive (Kuehn et al., 2018) and attentional (Puckett et al., 2017) factors to shape and/or consolidate cortical functional architecture. We, therefore, predicted that artists’ toe representation (in both the foot and hand areas) would resemble the canonical hand representation, i.e., more “hand-like” in its pattern, compared to controls. This should occur primarily for the artists’ dexterous foot, due to its striking skill, but perhaps also for the stabilizing foot that benefits from increased and specialized usage.

Each foot artist was separately compared to a group of typically developed and age-matched controls (using stringent tests developed specifically for case-study comparison, namely, Crawford t tests (Crawford and Howell, 1998), allowing for test-retest verification of the results. Results for the stabilizing foot are presented as supplementary figures.

## Results

### Hand-like Foot Use in Artists’ Daily Life

An assessment of the artists’ daily motor repertoire by using qualitative questionnaires revealed highly frequent and exceptional toe dexterity (Figures 1A–C; Video S1; for full results see Table S1). These measures indicated that artists performed the vast majority of daily life tasks with their feet (motor activity log [MAL]; see Makin et al., 2013; artist 1: 80%, artist 2: 76%) and with ease (MAL difficulty rating: rating “not difficult”; artist 1: 80%, artist 2: 66%). They both showed clear laterality preference (artist 1: −100, artist 2: +83; as assessed using an adaptation of the classical handedness questionnaire, Oldfield, 1971), and they have used almost all typical tools with their lower limbs (Tool Use Questionnaire, see Striem-Amit et al., 2018; artist 1: 88%, artist 2: 90%).

Video S1. Demonstration of Foot Artist’s Foot Dexterity, Related to Figure 1

### Somatotopic Toe Selectivity in Artists but Not Controls

**Figure 2:**
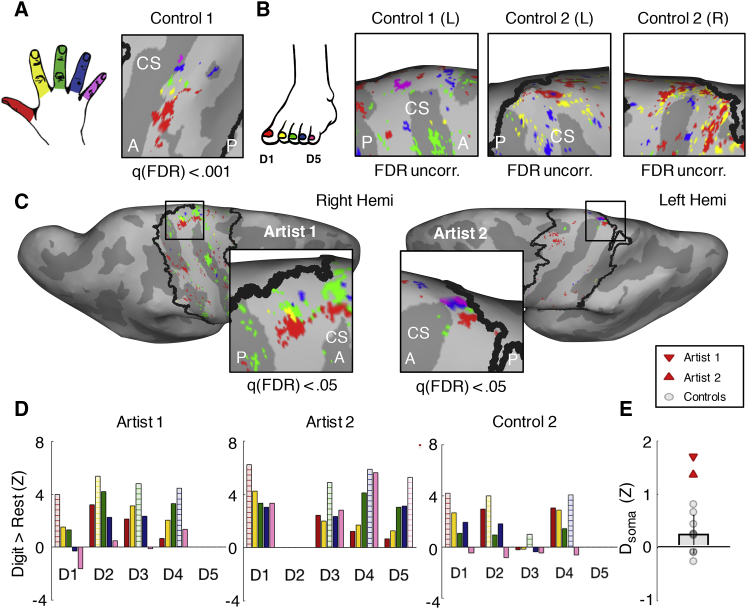
Toe Maps in the Foot Area (A and B) Univariate selectivity maps (each digit versus all others contrasts) are presented for (A). The hand of one control participant (as a visual reference for “hand-like” maps; lateral view) and for the foot of the same control (dorsal view) (B), and the feet of the median control (according to somatotopy scores, see (D). Controls did not show topographic toe maps (even without FDR correction; see full results in Figure S1). (C) Selectivity maps of the artists’ dextrous foot (dorsal view) at the medial aspect of the postcentral gyrus contralateral to the dextrous foot, following the medio-lateral somatotopic organization characteristic of hand maps. Values are FDR corrected (see Table S3). (D) Mean activity for each digit (versus rest) in digit-specific clusters (D1–D5, correspond to big-to-little toe) shows topographic organization for the artists’ dextrous foot but not for controls. Digits used to define each cluster are colored using horizontal stripes. When no significant cluster was found for a digit, the corresponding space has been left empty (e.g., artist 1, D5). Control results were averaged across hemispheres (see full results in Figure S2). (E) Somatotopy score (D_soma_; mean activity differences between neighboring digits [of each cluster’s strongest digit] and other digits) is higher for both artists than for controls (n = 9, averaged across hemispheres). The SD for control scores is indicated by a vertical line. See also Figures S1 and S2 and Table S3.

To describe somatotopic inter-toe selectivity, we used univariate selectivity contrasts between each digit and the average of the other four. As illustrated in Figure 2A, a typical hand map consists of digit-selective clusters, progressing from thumb (red, laterally) to little finger (pink, medially) (Sanchez-Panchuelo et al., 2012). Similarly, both artists showed clear digit-selective clusters for 4 out of 5 digits of their dexterous foot, located in the medial wall of the contralateral postcentral gyrus (Figure 2C). Visually, the toe maps mimicked those identified in non-human primates in terms of gross macroscopic organization and location on medial aspect of the postcentral gyrus (oriented medio-laterally) (Liao et al., 2016).

Using the same thresholds (q(false discovery rate [FDR]) < 0.05), we were unable to observe clear topographic arrangement elsewhere in the sensorimotor strip. Using a more lenient threshold (Z > 2.3), we also found similar maps for the artists’ stabilizing foot, similarly following medial-lateral D5-D1 somatotopic ordering (Figure S1). When examining each of the control participants’ maps separately (n = 18), even using a minimal threshold, we only identified one potential rudimentary map (C05; left hemisphere), with selectivity for 3 toes. No other consistent toe selectivity was identified in controls (Figures 2B and S1). These findings provide a preliminary demonstration of somatotopic toe maps, primarily for the artists’ dexterous foot.

### Somatotopic Toe Overlap in Artists but Not Controls

We next examined somatotopic overlap by investigating the activity profile within a given digit-selective cluster (e.g., D1 cluster) to the non-preferred digits (e.g., D2–D5). Somatotopic overlap is defined as greater activity for directly adjacent digits versus non-adjacent digits (Kolasinski et al., 2016). Both artists showed somatotopic gradients of activity levels (Figure 2D; see Figure S2 for individual control plots). To quantify somatotopy in the univariate analysis, we calculated the activity difference between directly adjacent digit(s) and non-adjacent digits (Figure 2E). The score for the artists’ dexterous foot was high (artist 1: 1.69, artist 2: 1.36), indicating a somatotopic activity gradient, whereas, in controls, the ratio was not significantly different from zero, although it was trending (mean rho [M] = 0.24, SD = 0.36, SEM = 0.12; one sample t test, t(8) = 1.99, p = 0.082). A direct comparison revealed somatotopy was greater for artists compared to controls (artist 1: t(8) = 3.87), p = 0.005; artist 2: t(8) = 2.98, p = 0.018; see Figures S2 and S3 for similar results for the artists’ stabilizing foot). This analysis indicates enhanced somatotopy of the artists’ toe-map activity patterns.

### Hand-like Inter-toe Activity Patterns in the Foot Area of Artists

**Figure 3:**
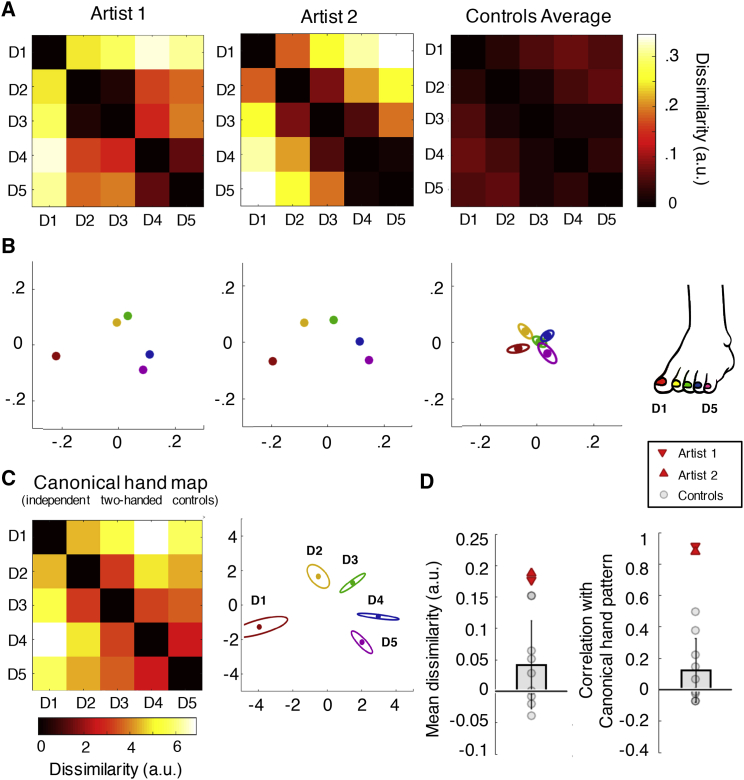
Toe Activity Patterns in the Foot Area by Using Representational Similarity Analysis (A) Representational dissimilarity matrices (RDMs) for the artists’ dextrous foot and controls (average, n = 9) showing dissimilarity (lighter colors) versus similarity (darker colors) of inter-digit multivariate representation patterns (Mahalanobis distances). (B) Dissimilarity between digit activity patterns represented by spatial distance, i.e., two-dimensional projections of the RDM, by using multi-dimensional scaling. Individual digits are presented in different colors (see color key in C); ellipses reflect control SE after Procrustes alignment. (C) As a visual reference, the matrix and projection for a canonical hand in the hand area of typically developed participants (average). (D) Mean RDM dissimilarity values and correlations between toe RDM in the foot area and the canonical hand RDM for individual controls (gray) and the artists (red). Both artists showed a stronger correlation between canonical hand RDM and their toe RDM compared with controls, indicating more hand-like patterns. Error bars represent SD across controls. All other abbreviations are as described in the Figure 2 legend. See also Figure S3 and Table S2.

The results described above are threshold- and cluster-size dependent. To examine fine-grained, threshold-free toe representation, we studied the organization of multivoxel activity patterns. We assessed inter-digit (dis)similarity, producing a 5 × 5 representational dissimilarity matrix (RDM; 1 cell per digit pair contrast; Figure 3A), previously extensively studied for hand representation (Ejaz et al., 2015, Wesselink et al., 2019, Yokoi et al., 2018).

In the dexterous foot’s region of interest (ROI; see STAR Methods), we found no differences in overall activity levels between each of the artists and the controls (averaged across all toes; artist 1: t(8) = 0.36, p = 0.363; artist 2: t(8) = 0.88, p = 0.404). To investigate the representational structure of activity in this ROI, we first examined the inter-digit RDMs (Figures 3A and 3B). The average inter-digit dissimilarity values (calculated per participant) tended to be higher for artists compared to controls (artist 1: t(8) = 2.01, p = 0.079; artist 2: t(8) = 2.18, p = 0.06; Figure 3D), suggesting digit representations were more individuated in artists, but this did not reach significance. Critically, however, we wished to examine the organization of information within this area to determine whether the activity patterns reflected the predicted hand organizational structure, i.e., was toe representation more hand-like for the artists than for controls?

We quantified hand-like representation by correlating the foot area’s representational structure (RDM) with a canonical hand RDM, obtained from an independent group of typically developed individuals by using similar imaging parameters (Wesselink et al., 2018; see Figure 3C). Both artists showed a high correlation (artist 1: rho = 0.915, artist 2: rho = 0.879), resulting in a significantly more hand-like pattern compared to controls (controls, mean rho = 0.125, SD = 0.20; t(8) = 3.67, p = 0.006 and t(8) = 3.50, p = 0.008 for artists 1 and 2 versus controls, respectively; Figure 3D). This result supports the above observation of a hand-like toe map in the dexterous foot region of artists but not controls (see Table S2 for supplementary results in control ROIs). The stabilizing foot of both artists was also more hand-like than controls (see Figure S3).

### Hand-like Inter-toe Activity Patterns in the Hand Area of Artists

**Figure 4:**
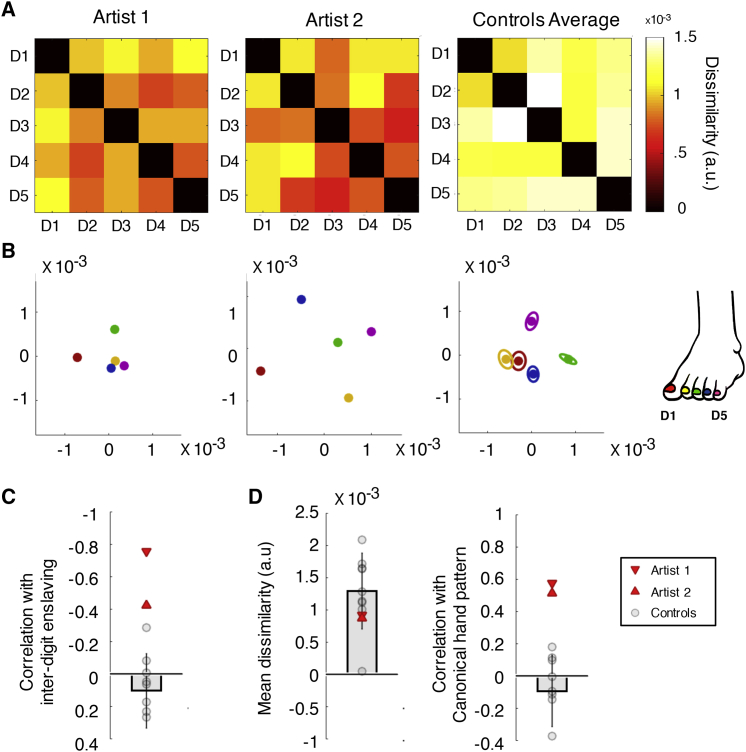
Toe Activity Patterns in the Hand Area by Using Representational Similarity Analysis (A) Representational dissimilarity matrices (RDMs). (B) Spatial two-dimensional representation of dissimilarity. (C) Correlations between toe RDM in the hand area and toe individuation matrix during free movement (kinematics; Figure 1D) were stronger for both artists than controls. (D) Mean RDM dissimilarity values, and the correlation between toe RDM in the hand area and the canonical hand RDM. Both artists showed greater hand-like correlation compared with controls (n = 9). All other details are as in Figure 3 legend. See also Figure S3.

We next examined toe representation in the artists’ (missing) hand area, contralateral to the dexterous foot. Contrary to previous reports (Stoeckel et al., 2009, Striem-Amit et al., 2018, Yu et al., 2014, Yu et al., 2006), we found no difference in toe activity levels in artists versus controls (artist 1: t(8) = 0.78, p = 0.456; artist 2: t(8) = 1.03, p = 0.333; see Figure S2 for whole-brain contrasts and Discussion). Inter-toe dissimilarity (average RDM) also did not differ significantly (both artists versus controls, p > 0.553; Figure 4D). Importantly, when examining the hand area representational structure, we found that the dexterous foot’s toe representation was significantly more hand-like in both artists compared to controls (artist 1: rho = 0.576; artist 2; rho = 0.515; controls: mean rho = −0.092, SD = 0.22). This was demonstrated by a significantly greater correlation between the foot and the canonical hand RDMs in both artists versus controls (artist 1: t(8) = 2.85, p = 0.022; artist 2: t(8) = 2.88, p = 0.020; Figure 4D). This result demonstrates that toe-related activity in the missing hand area is at least loosely organized and that this organization mirrors native hand organization features.

### Greater Correlation between Toe Representation and Toe Kinematics in Artists

We next studied correspondence between brain activity and individuals’ ability to individuate their toes during free movement. For this purpose, we recorded inter-toe kinematics, asking participants to move one (“instructed”) digit while keeping the other (“non-instructed”) digits still, producing a 5 × 5 inter-toe motor enslaving (anti-independence) matrix (Figure 4D). This kinematic matrix was correlated with the toe (RDM) matrices identified in the foot and hand areas.

Despite the artists’ exceptional foot dexterity, their net ability to individuate toes in free movement, measured using the mean kinematics matrix, did not differ from controls (n = 14; artist 1: t(14) = 0.24, p = 0.816; artist 2: t(14) = 0.72, p = 0.485; full details of the task design, analysis, and unpublished results can be found in https://osf.io/4q8cs/). Importantly, the organization of the artists’ kinematics matrices with respect to brain organization was distinct from controls. Specifically, the artists’ respective inter-digit enslavement patterns (as captured using kinematics) correlated more strongly with their brain activity pattern (toe RDM pattern), compared to controls (perhaps driven by D1 individuation in both brain and behavior, see Figure 1D). This resulted in significant differences between either artist versus controls in the hand area contralateral to the dexterous foot (controls, mean rho = 0.103, SD = 0.23; artist 1: rho = −0.758, t(8) = −3.96, p = 0.004; artist 2: rho = −0.424, t(8) = −2.42, p = 0.042; see Figure 4C). A similar pattern (albeit not consistently significant, as artist 2 was only showing a trending effect) was found when comparing kinematics with the toe RDMs of the foot-area (controls mean rho = −0.168, SD = 0.28; artist 1: rho = −0.867, t(8) = −2.66, p = 0.029; artist 2: rho = −0.709, t(8) = −2.06, p = 0.073). This analysis demonstrates that the patterns of foot movement are more strongly reflected in brain activity patterns of the artists, supporting a role for usage in shaping some aspects of the organized toe representation (alongside other sensory and high-order usage-related factors, see Discussion).

### Enhanced Tactile Perception for the Toes of Artists

Finally, we tested spatial and temporal measures of tactile perception, previously shown to benefit from increased cortical magnification (Jenkins et al., 1990a, Jenkins et al., 1990b, Recanzone et al., 1992). Both artists showed superior tactile spatial acuity with their dextrous foot compared with controls (n = 21), as demonstrated in greater intra-toe orientation discrimination (t(20) = 2.27, p = 0.033 for each of the two artists) and inter-toe temporal order judgements (artist 1: t(20) = 3.37, p = 0.003; artist 2: t(20) = 2.50, p = 0.021) but no differences in tactile temporal frequency discrimination (artist 1: t(19) = −0.08, p = 0.938; artist 2: t(19) = −0.28, p = 0.783; Figure 1G). This suggests improved spatial (but not temporal) tactile perception, perhaps due to incidental sensory “training” through usage (Dempsey-Jones et al., 2016, Harrar et al., 2014) or from changed brain organization resulting from a combination of deprivation and training (Dempsey-Jones et al., 2019, Grant et al., 2000, Heller, 1989; although see Stoeckel et al., 2004 for worsening of light touch in bilateral amelics).

## Discussion

Here, we asked whether we could identify representational features of functional brain organization that do not exist in controls but do in foot artists, demonstrating an extreme form of habitual behavioral adaptation. First, we identified somatotopic toe maps in artists but not typically developed two-handers. We also found multivariate toe activity patterns showed a greater correspondence with the canonical hand organization (i.e., were more hand like) in artists than controls. This was seen for both feet in the foot region and in the (missing) hand area for the dextrous foot only. Finally, artists had a stronger association between brain and kinematic toe individuation patterns for their dexterous foot, as well as heightened spatiotactile toe perception compared to controls.

### Complexity in Bodily Experience

As stated in the introduction, the dramatically divergent profile of the artists’ toe behavior likely supports their detailed toe organization in the brain. This habitual behavior extends well beyond extreme motor skill (e.g., striking compensatory behaviors and tool use with the feet and toes, including feeding with cutlery, writing, drawing, and computer use; see Figures 1A–1C; Table S1). For example, the artists’ lives spent largely without enclosed footwear will have dramatically altered inter-toe cutaneous stimulation, previously shown to modulate finger selectivity in BA3b (Jenkins et al., 1990a, Wang et al., 1995). Visual experience of touch (Kuehn et al., 2018) and spatial attention to tactile stimulation (Puckett et al., 2017) both induce digit-selective activity in SI and likely also play a role in the generation and maintenance of the artists’ somatotopic toe maps. This range of motor, sensory, attentional, and cognitive factors accords with the presence of the artists’ toe maps in their foot area for both feet (see below for further discussion of foot laterality).

Despite a clear superiority in their toe behavioral repertoire (e.g., in writing and drawing and other tasks detailed in the qualitative analysis), we did not find enhanced individuation of single-toe movement in artists compared to controls by using our kinematic task. As such, active toe individuation may not be a critical feature that defines the artists’ brain and behavior (N. Ejaz et al., 2016, Soc. for Neurosci., conference) for similar results in musicians). Indeed, the natural statistics of action (Ingram et al., 2008), which have been identified as the critical link between motor behavior and sensorimotor brain organization (Ejaz et al., 2015), encompass complex inter-digit synergic coordination and the resultant sensory and cognitive inputs. Thus, although our task captured rudimentary aspects of motor control that were relevant to functional brain organization in the artists (as indicated by greater brain-behavior correlation versus controls), brain activity may be more completely explained by habitual daily behavior (see Video S1).

### Latent Genetic Template

Medial-to-lateral D5–D1 toe somatotopy has previously been documented in macaques (Liao et al., 2016, Nelson et al., 1980), although toe selectivity may be weaker than that for fingers (Hashimoto et al., 2013). Given the qualitative similarities in terms of gross macroscopic organization and brain location between the toe map of non-human primates and our artists, it may be that humans are born with a genetic predisposition for developing toe maps, given sufficiently complex sensorimotor input (Krubitzer and Prescott, 2018). In “non-foot-users,” due to the lack of relevant experience, individualised toe representation would fail to develop from its genetic template. Alternatively, toe somatotopy may be realized at birth but deteriorates due to habitual cooperative toe movement and imprecise tactile input (as seen in rodents’ barrel cortex following whisker trimming (Feldman and Brecht, 2005). The latter theory raises the interesting prediction that somatotopic toe mapping may exist in human infants.

### Bodily Experience in the Critical Period

Our previous work has emphasized that timing of sensorimotor experience is critical for somatotopic organization. For instance, hand representation persists in arm amputees despite decades of non-use of their missing hand (Kikkert et al., 2016, Wesselink et al., 2019) (see also Bruurmijn et al., 2017, Flesher et al., 2016). Conversely, people with congenital unilateral limb loss show no organized finger representation for their missing hand (Wesselink et al., 2019). Thus, it appears that early life bodily experience is necessary for the development but not maintenance of somatotopic maps. Also, supporting the timing of experience, some individuals who lose their upper limbs in early childhood were reported to develop extreme toe motor skills akin to those seen in congenital limb loss (Yu et al., 2014, Yu et al., 2006). In contrast, it has not been possible thus far to train typically developed adults to improve toe individuation (Friedman and Goodman, 2016, Motor Control 2016 - Bridging motor control and biomechanics, conference). Non-lateralised foot-use in early life may also explain the artists’ toe maps in the foot area for both feet. Dexterity develops over an extended period over childhood (Michel et al., 2016), meaning semi-skilled behavior of both the left and right feet in early childhood may have shaped toe somatotopy before the artists developed a dextrous foot preference. Accordingly, finger representation in the typically developed hand area shows no clear differences for the dexterous versus stabilizing hand (Barnsley and Rabinovitch, 1970, Boakye et al., 2000, Steenhuis, 1999, White et al., 1997).

### Foot Representation in the Hand Area

Previous studies reported lateral activity clusters in the cortical hand area, in addition to the medial foot area, in response to toe movement in individuals with bilateral congenital amelia (Stoeckel et al., 2009, Striem-Amit et al., 2018; see also Hahamy et al., 2017, Hahamy and Makin, 2019) and passive toe stimulation in individuals with early childhood arm amputation (Yu et al., 2014, Yu et al., 2006). Common to all such studies is the usage of univariate fMRI activity, where net changes in signal amplitude are averaged over a ROI. This method solely informs on whether foot-related brain activity is statistically increased in the missing hand area, e.g., relative to baseline, other body parts, or a control group. As such, these previous studies are unable to determine whether such activity represents functional reorganization supporting sensorimotor foot processing (such as demonstrated using transcranial magnetic stimulation [TMS] [Stoeckel et al., 2009]) or simply non-functional activity changes subsequent to the absence of hand-inputs (e.g., due to upstream reorganization [Kambi et al., 2014]), aberrant signaling (Vaso et al., 2014), changed local metabolism (Cirstea et al., 2017), or decreased inhibition (Hahamy et al., 2017)].

Multivoxel pattern analysis allows us to decipher the information content underlying changed activity or even in the absence of activity changes (Haynes and Rees, 2005, Kamitani and Tong, 2005). As this analysis is also able to identify consistencies in representational structure when activity levels are different (Arbuckle et al., 2019, Diedrichsen et al., 2013), it provides a measure dissociated from univariate activity and is arguably better suited to identify representational changes. As such, it can uncover persistent or changed brain organization, which might be buried under the net activity changes producing remapping or even non-functional activity changes (see Bodily Experience in the Critical Period). This approach allowed us to determine whether the deprived brain area (whether undergoing clear remapping or not) actually contains representational features that may be relevant for the enhanced foot use repertoire of the artists.

Our multivoxel pattern analysis revealed that the missing hand area contralateral to the dextrous foot shares representational features with a canonical hand representation, beyond what is found in controls. We previously speculated that representations in the missing-hand territory of congenital one-handers can be flexibly distributed to body parts that share the same functional utility as the missing hand (Hahamy et al., 2017) (although others have argued against the role of altered bodily experience in shaping topography [Striem-Amit et al., 2018]). According to our adaptive-usage account, synergistic inputs that more closely mimic the missing hand’s function across body parts and sensory systems (e.g., mouth-foot interaction in feeding with the feet; visuomotor interactions when manipulating objects) may consolidate preferentially in the missing hand territory, due to large-scale connectivity architectural constraints (Graziano and Aflalo, 2007, Mahon and Caramazza, 2011). Our current results lend empirical support for the relationship between compensatory behavior and brain reorganization, as demonstrated by the correlation between activity patterns in the missing hand area and the artists’ ability to move their toes independently in motor action. This may indicate the (typical) hand territory need not necessarily represent the hand per se but rather any other body part that can mimic the missing hand’s functionality.

### Conclusions

We identified key features of canonical hand representation in the foot representation of handless individuals who use their feet to substitute missing hand function that considerably exceeds that seen in two-handed controls. Given that it has not yet been possible to improve foot dexterity with training in two-handed adults (Friedman and Goodman, 2016, Motor Control 2016 - Bridging motor control and biomechanics, conference), it remains an outstanding challenge to delineate the competing roles of genetics, behavior, and developmental plasticity in shaping this distinct functional organization.

## STAR★Methods

### Key Resources Table

**Table:** 

REAGENT or RESOURCE	SOURCE	IDENTIFIER
**Deposited Data**		

Acquired and analyzed data	This paper	https://osf.io/4q8cs/, https://doi.org/10.17605/OSF.IO/4Q8CS
Representational dissimilarity matrices for controls’ hands	Wesselink, et al., 2018	https://osf.io/gmvua, https://doi.org/10.17605/OSF.IO/GMVUA

**Software and Algorithms**		

FSL	Smith et al., 2004	https://fsl.fmrib.ox.ac.uk/fsl/fslwiki/
Freesurfer	Freesurfer	https://www.humanconnectome.org
FSL RSA	Rsatoolbox, version 20fbe05	https://github.com/ronimaimon/rsatoolbox

### Lead Contact and Materials Availability

Further information and requests for resources should be directed to and will be fulfilled by the Lead Contact, D.B. Wesselink (d.wesselink@ucl.ac.uk).

No new materials have been generated by this study.

### Experimental Model and Subject Details

#### Human subjects

Foot artists (age 55 and 56, both male) completed all components of the testing procedures. The control group (n = 21) were typically developed and age-matched (see Table S4 for full details of all participants). Fifteen controls completed tactile and motor testing (age, M = 56.86, 8 males), of which 9 were also tested in the fMRI protocol (age, M = 54.67, 4 males). A further 6 controls underwent tactile testing only (age, M = 50.67, 3 males). Ethical approval was granted by the NHS National Research Ethics service (Ref: 10/H0707/29) and written informed consent was obtained from all participants prior to the study.

### Method Details

#### General procedure

For participants who completed all three components of the study (sensory, motor, fMRI), data was collected over three separate sessions, completed on different days. Half the participants completed the sensory tasks then imaging, and the other half performed these tasks in reverse. The motor task was collected on a separate session.

All fMRI analysis was restricted to the primary sensorimotor cortex (except for the whole-brain contrasts shown in Figure S2).

#### Imaging procedures

##### Task design and instructions

All participants underwent a passive toe stimulation experiment (main experimental task), an active body-part movement task (localizer task) and a structural scan. A passive rather than active paradigm was chosen since individuated toe movements are impossible to produce consistently in controls (as demonstrated in our kinematics task, see Figure 1D); though both active and passive paradigms have been effective in demonstrating digit topography in the hand (Kolasinski et al., 2016, Sanchez-Panchuelo et al., 2012) and body-part remapping (Hahamy et al., 2017, Stoeckel et al., 2009, Striem-Amit et al., 2018, Yu et al., 2014, Yu et al., 2006).

The main experimental task involved repeated passive tactile stimulation of the foot digits at approximately 1 Hz rate. The design structure was adapted from Diedrichsen et al. (2013). All participants underwent 4 runs per foot. Each run consisted of 3 repetitions of 12 s blocks of stimulation per digit interspersed with 4 rest blocks. Tactile stimulation was administered by an experimenter receiving auditory cues regarding the stimulation order and timing using Presentation software (version 0.70, http://www.neurobs.com). Each individual digit pad was touched with force sufficient to minimally deflect the digit. The tactile stimulus covered most of the pad of D2-D5; for D1, the entire pad could not be stimulated at once, and stimulation location was varied to achieve maximum tactile coverage across trials. In order to help the participants focus on the stimulation and maintain attention, they received minimal visual feedback during the block regarding which toe was stimulated (one of five circles on screen would flash when its corresponding digit was stimulated).

Furthermore, the participants were instructed to “notice” (but not respond physically) the incidence of ‘catch trials’, in which 1 tap was replaced by a rapid double tap (not reflected in the visual feedback; the remaining trials in the block did not change). There were five catch trials per run (1 per digit). Each run contained 5 catch trials (1 per digit). Prior to analysis we confirmed general activity to passive toe stimulation (average of all digits versus rest) was equivalent between artists and controls, in both the foot and hand areas (see ROI definition below). This supports consistent brain responses to stimulation, justifying direct comparison. Critically, our primary measure of interest, inter-digit relationships reflected in multivariate patterns (see below) is largely invariant to general activity differences (Arbuckle et al., 2019; see also Figure 5 in Berlot et al., 2019).

The active localizer task involved movement of the left or right foot (all toes flexion/extension), mouth (lip pursing) and rest blocks. Each condition was repeated five times (12 s blocks) at roughly 1Hz frequency. Participants were visually instructed to move using text.

As part of an extended protocol, scanned participants also underwent two additional scans that were not included in the manuscript, and subsequently, will not be discussed here. These tasks involved a resting state scan and passive stimulation task using an alternative, phase-encoding protocol.

##### MRI acquisition

MRI measurements were obtained using a Siemens 7 Tesla Magnetom scanner with a 32-channel head coil. fMRI data was acquired using Multi-Band EPI (acceleration factor 2) with a limited (horizontal) field of view, capturing to superior portion of the cortex: 56 slices with a 192 × 192 in-plane field of view. The following parameters were used: spatial resolution: 1mm isotropic; TR: 2000ms; TE: 25ms; FA: 85 deg; phase partial Fourier: 6/8; and PE acceleration factor: 3. Fat suppression was done by CHESS.

Anatomical T1-weighted (MPRAGE) images consisted of a whole-brain single image with a 1 mm isotropic resolution. The following parameters were used: FA: 7 deg; TI: 1050 ms; TE: 2.82 ms; TR: 2200 ms. Fat suppression was done by means of water excitation.

##### fMRI pre-processing and low-level analysis

Data pre-processing, general linear model (GLM) analysis and cortical surface reconstruction were implemented using FSL (https://fsl.fmrib.ox.ac.uk/fsl/fslwiki/) and Freesurfer (http://www.freesurfer.net). Connectome Workbench (https://www.humanconnectome.org) was used for visualization on the cortical surface. Additional scripts were written in UNIX or MATLAB (https://github.com/ronimaimon).

Each run was pre-processed using FEAT 6.0, and included: motion correction, brain extraction, high-pass temporal filtering (100sec) and minimal spatial smoothing using a 1mm FWHM (full width at half maximum) Gaussian kernel (see Hendriks et al., 2017 for the effect of smoothing on MVPA). The results from the motion estimates were inspected for excessive subject motion: no run contained more than 1 mm of relative displacement. Co-registration was carried out using FLIRT), and manually adjusted for accuracy. Anatomical T1 images were used to reconstruct the pial and gray matter surfaces using Freesurfer.

A voxel-wise GLM was applied to each of the task runs, as implemented in FEAT. The design was convolved with a double-gamma function and its temporal derivative. From the main task, 11 contrasts were defined per run: each individual digit versus rest (for multivariate analysis), each individual digits versus all other digits (for univariate analysis) and all digits versus rest. The voxel-wise estimates were averaged across the four runs for each participant using a fixed effects model. For the active (localizer) task, contrasts were defined for each body part versus rest, each foot versus mouth, and mouth versus the average of both feet.

##### Regions of interest

ROIs for each of the foot and hand areas, as well as the mouth, were defined on the cortical surface. The anterior-posterior boundaries were constrained to BA 1, 2, 3a, 3b, 4a, 4p using non-thresholded single-subject parcellations from Freesurfer (http://www.freesurfer.net). For the foot region, ROIs were defined for each participant separately and within each hemisphere by applying the contrast ‘contralateral foot versus mouth’ (from the functional localizer described above) using threshold Z > 5 and choosing the largest contiguous cluster. For the hand region, we used a mask from a previous study (Hahamy et al., 2017) based on group hand movement versus rest. This mask was then converted to individual’s native anatomical space.

##### fMRI analysis: Univariate analysis

To identify dexterous digit maps within the primary sensorimotor cortices, contrasts for each individual digit versus all other digits were first corrected for multiple comparisons using family-wise error (Z > 2.3, cluster determinant p < 0.05; whole brain). Note that this minimal threshold was used in order to minimize Type II errors in controls i.e., missing subtle but present maps. For artists (Figure 2), maps were additionally thresholded based on a false discovery rate (FDR) criterion q(FDR) < 0.05 (accounting for voxel-wise error within the sensorimotor strip) for each contrast separately. This additional step was taken to minimize Type I errors for the reported maps. The Z-values corresponding to the thresholds for the results corrected for FDR are reported in Table S3. Note that the latter step was wavered in Figure S1. Resulting maps were projected onto the participant’s native inflated surface for visualization.

To quantify the degree to which the digit maps followed a somatotopic organization, co-selectivity was assessed. First, within each foot area (described above), for each digit, a sub-region was created from all voxels selective to that digit (according to the contrast above). Within each sub-region, we calculated the difference in activity between the digit(s) directly adjacent to the most strongly active digit (i.e., the one used to define the sub-region) and the digits further away. These values were averaged across digits, ignoring any digit no voxels were selective for, resulting in a somatotopy score (D_soma_), where a high score indicates somatotopic co-activation. The scores for controls were averaged across hemispheres.

##### Representational similarity analysis

To assess the structure of digit-related activity, the similarities between single-digit activity patterns (derived from the pre-processing pipeline as elaborated above) were calculated for each foot in the contralateral hemisphere over the foot and hand ROIs. For each digit pair, the distance metric was the Mahalanobis distance (Ejaz et al., 2015, Nili et al., 2014), cross-validated over each possible pair of the 4 runs and then averaged. Because of cross-validation, the expected distance between identical conditions is 0 (and will therefore be negative half the time) (Nili et al., 2014). The resulting inter-digit distances were arranged in a dissimilarity matrix (RDM), and two measures were taken: individuation, i.e., the average inter-digit distances comprising the RDM; and the shape of the representational structure, i.e., the Spearman correlation between the individual RDM and a group inter-digit RDM for finger representation in the hand area (taken from Wesselink et al., 2018). In brief, this hand matrix contained the group average representational dissimilarity values in S1 between the 5 digits of the dominant right hand of two-handed controls (N = 9). The values were acquired at 7T using an active single-digit tapping task. The analysis pipeline was similar to above. This hand matrix was taken to represent the canonical activity pattern for the hand. Additionally, the RDM was (Spearman) correlated with the individual’s dissimilarity values from the toe individuation kinematics matrices (see below). We used Spearman (rank) correlations, because parametric statistics may be problematic for RDMs containing only 10 unique cells (Bishara and Hittner, 2012).

As an aid to visualize the representational structures, we also used classical multidimensional scaling (MDS). MDS projects the higher-dimensional structure into a lower-dimensional space, while preserving the inter-digit dissimilarity values as well as possible (Borg and Groenen, 2005). MDS was performed on data from individual participants and (if applicable) averaged after Procrustes alignment (without rescaling) to remove arbitrary rotation induced by MDS. The between-subject standard error (in two dimensions, parallel and orthogonal to the direction of greatest error) is depicted by ellipses.

#### Motor Tasks

##### Experimental procedures and instructions

We assessed the artists’ compensatory motor skill through quantitative and qualitative measures. Full details of the quantitative measures (questionnaires) is available in Table S1. The Motor Activity Log is a clinical questionnaire previously validated to assess use of residual limbs and prostheses (e.g., Makin et al., 2013) – here adapted for lower limbs. We used an adaptation of the Edinburgh Handedness Questionnaire (Oldfield, 1971) to assess preferred foot for performing 10 daily tasks. The tool use questionnaire assessed how often 42 different tools were used for their typical function with the upper/ lower limbs and mouth.

For quantitative assessment we used a kinematic motor task assessing motor individuation of the toes. Magnetic motion sensors (Ascension trakSTAR) were attached to the nail of each of the toes. An additional reference sensor was taped on the top of the foot. Participants were instructed to move one toe up and down at 1Hz rate (using auditory cures) while keeping all other toes still (Figure S4). There were two blocks per toe (10 blocks in total, order randomized). Please see https://osf.io/4q8cs/ for the full experimental protocol.

##### Calculation of digit individuation

As our measure of individuation, we calculated the Spearman correlation of movement velocity of the instructed and non-instructed toes. Negative correlations were recoded as their absolute values because these also indicate enslaving (though negative correlations were minimal, and when present were of low value – so there was no difference in the pattern of results when using raw correlational values). The resulting correlations were arranged into a 5 × 5 matrix with each cell representing the correlation of one possible pairing of toes (rows represented instructed toe conditions one to five, and columns the non-instructed toes; Figure 1D).

#### Sensory Tasks

The foot stimulated was the dextrous foot for artists, or one selected (dominant) foot of the controls (typically right; see Table S4 for full details).

##### Two-point orientation discrimination

This task involved presentation of a two-pronged instrument (spacing 2,3,4 and 5mm) to the big toe (D1) pad. These prongs were oriented either across or down (trial-by-trial), with respect to the proximal-distal toe axis, using descending spacing order (see Craig and Johnson, 2000 for advantages of this task over the traditional two-point discrimination). Participants verbally indicated the orientation at each trial. Proportion of correct trials were averaged as a measure of inter-digit tactile spatial acuity.

##### Temporal order judgements

Custom made vibrotactile stimulators (Dancer Designs) delivered two single, consecutive pulses (20ms) to either D1-D2 or D2-D3, one pulse per toe. The temporal distance between the two toes varied (inter-stimulus intervals (ISI) 30, 70, 100, 150, 200 and 300ms; 10 repetitions per ISI). Participants indicated which toe was stimulated first, by verbally reporting ‘left’ or ‘right’. The proportion of correct trials was used as the measure of inter-toe tactile spatial acuity.

##### Frequency discrimination

Vibrotactile stimulators delivered two consecutive 500ms pulses of vibrational stimuli (ISI 1sec) to D2 (as described above). Frequency was either the same across the two pulses or different. The original difference between the two frequencies was set to 16Hz, and an adaptive descending staircase was used to determine the frequency discrimination threshold over 45 trials. Participants reported whether the two frequencies were the same or different. Average staircase value (indicating frequency difference in arbitrary units) was used for comparisons between participants.

### Quantification and Statistical Analysis

Statistical analyses were done using MATLAB R2016a. For all tests requiring comparison of the artists to controls, we used two separate Crawford-Howell t tests to demonstrate test (artist 1), retest (artist 2) consistency. Crawford-Howell t tests were developed and validated specifically for case-study comparisons: (Crawford and Howell, 1998). To defend our results from type I errors, we only accepted results that were independently observed as significant in both case tests. Therefore, further correction for multiple testing was not required. All statistical comparisons between artists and controls were carried using 2-tailed tests. Since control participants have no dexterous foot, when the same measures were available from both feet these measures were averaged and submitted for group comparisons after verifying no significant differences across feet using paired t tests (though note the difference between sides for behavioral kinematic distances, i.e., raw individuation was trending at p = 0.092). For the sensory task, we selected the right foot for testing in the majority of participants. Two individuals were tested on the left foot for comparison, but scores were highly consistent and were, therefore, collapsed across feet. Matrices were compared using Spearman correlations. We used Spearman (rank) correlations, because parametric statistics may be problematic for RDMs containing only 10 unique cells (Bishara and Hittner, 2012).

In figures, bar plots indicate the group mean of the controls and the error bars indicate the standard deviation within the control group. Multi-dimensional scaling plots indicate the (2-dimensional) standard error using ellipses. In group comparisons, individual data points represent individual participants (rather than trials).

### Data and Code Availability

Datasets generated during this study are available at Open Science Framework (https://osf.io/4q8cs/).

## Supplemental Information

Document S1. Figures S1–S4 and Tables S1–S4

Document S2. Article plus Supplemental Information
