# Supplementary material for: Organized Toe Maps in Extreme Foot Users
Source: Cell Rep. 2019 Sep 10;28(11):2748–2756.e4. doi: 10.1016/j.celrep.2019.08.027 (PMC6899508; doi:10.1016/j.celrep.2019.08.027)
Supplement: Document S1. Figures S1–S4 and Tables S1–S4 [file mmc1.pdf]

Cell Reports, Volume *28*

Supplemental Information

Organized Toe Maps

in Extreme Foot Users

Harriet Dempsey-Jones, Daan B. Wesselink, Jason Friedman, and Tamar R. Makin

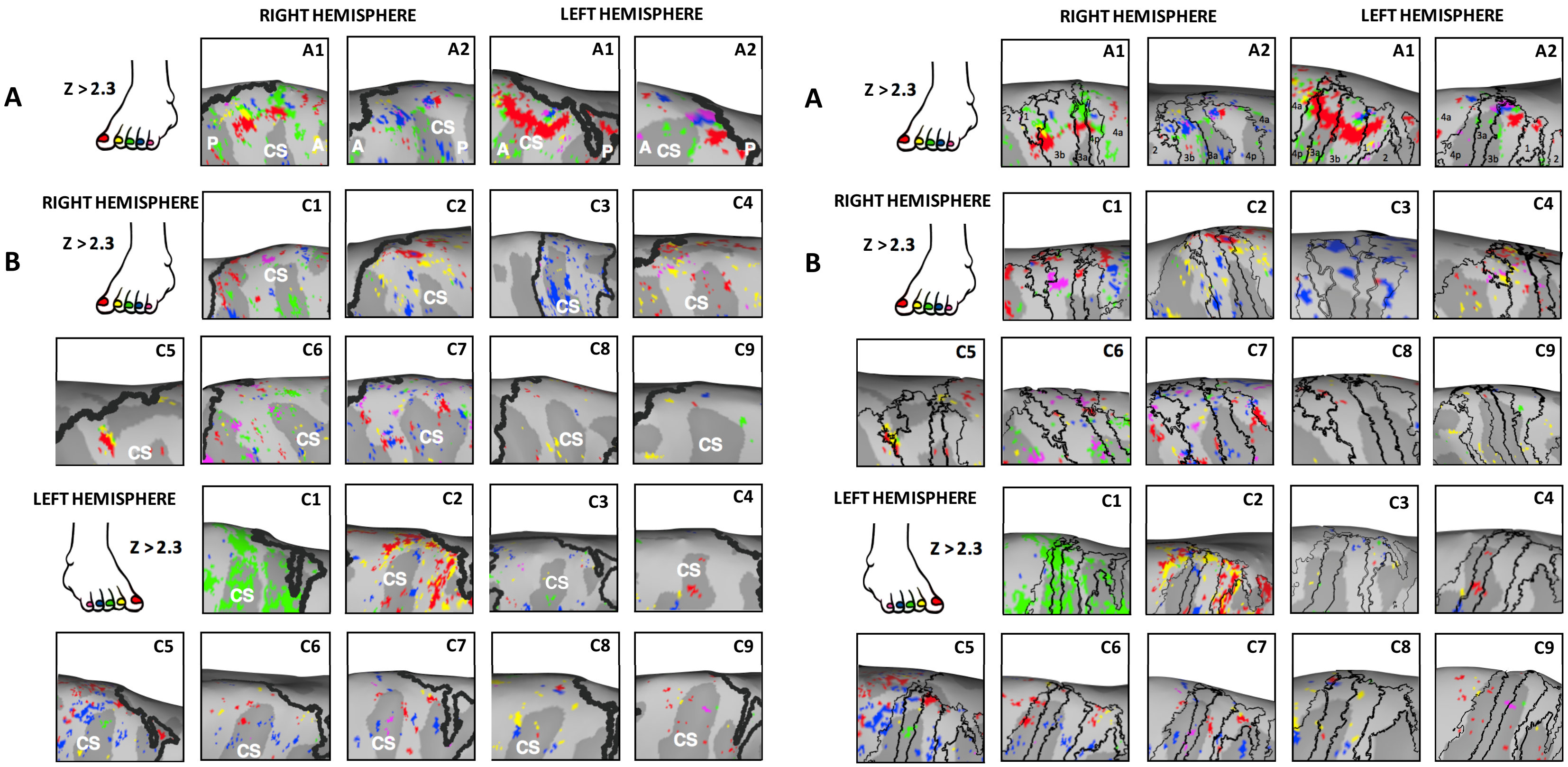

Figure S1, *Toe maps in the foot area*, related to Figure 2. Univariate contrast maps (activity for one digit vs. all others) illustrating selective activity to individual toes in the medial aspect of the central sulcus. Selectivity maps are presented for both hemispheres of artists and individual controls (showing activity for the contralateral foot). Left: Both artists displayed distinct clusters for 4-5 of their toes contralateral to both the dextrous foot (RH for artist 1 and LH for artist 2) and in Artist 1’ s stabilising foot, following the medio-lateral topographic organisation characteristic of hand maps, and of toe maps in monkeys. Overall, controls did not show consistent topographic foot maps in either hemisphere; though see C5 who shows selectivity for digits 2-4 in a generally topographic order on the anterior-posterior plane. All maps are shown on inflated brains; individual digits are presented in different colours (see colour keys in the side panels). Right: The area demarcations for Brodmann areas 2, 1, 3b, 3a, 4p and 4a are shown. Images may be reoriented for clarity.

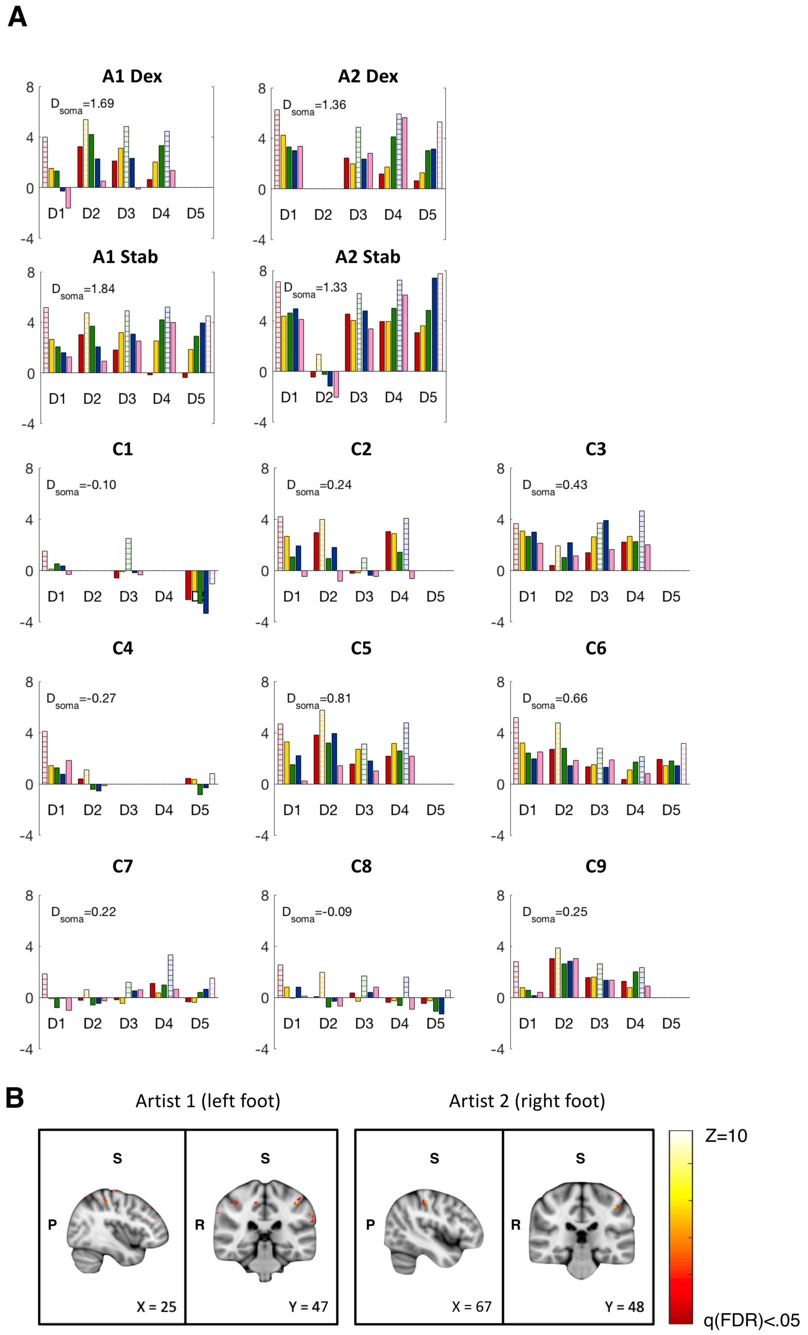

Figure S2, *Toe activity*, related to Figure 2. A) Selectivity graphs in Foot area (activity versus rest, in digit-specific clusters) illustrating somatotopic organisation (higher activity in digits neighbouring each cluster's strongest digit, than in digits further away; indicated by D_{soma} score). Somatotopy was more pronounced for artists' stabilising foot as compared to controls (artist 1 vs. controls, $t(8)=4.26$, $p=.003$; artist 2 vs. controls, $t(8)=2.90$, $p=.019$).Abbreviations: Dex: dextrous foot; Stab: stabilising foot. All other annotation as in Figure 2D. B) *Whole brain contrasts for toe activity in artists vs controls.* For each participant, an average activity map (across all toes) was generated for the whole brain (slab). Activity maps where then compared between each artist and the group of controls, using the Crawford-Howell t-tests, developed and validated specifically for case-study comparisons. The resulting group contrasts were corrected for multiple comparisons, using a false discovery rate q(FDR)<.05. Red clusters show areas of increased activity in the artists, compared to controls, relating to activity of the dexterous foot. Both artists showed increased activity in the postcentral sulcus, posterior to the central sulcus hand knob. This activity was bilateral for Artist 1 and contralateral for artist 2. No increased activity was observed in the proper S1/M1 hand area**.**

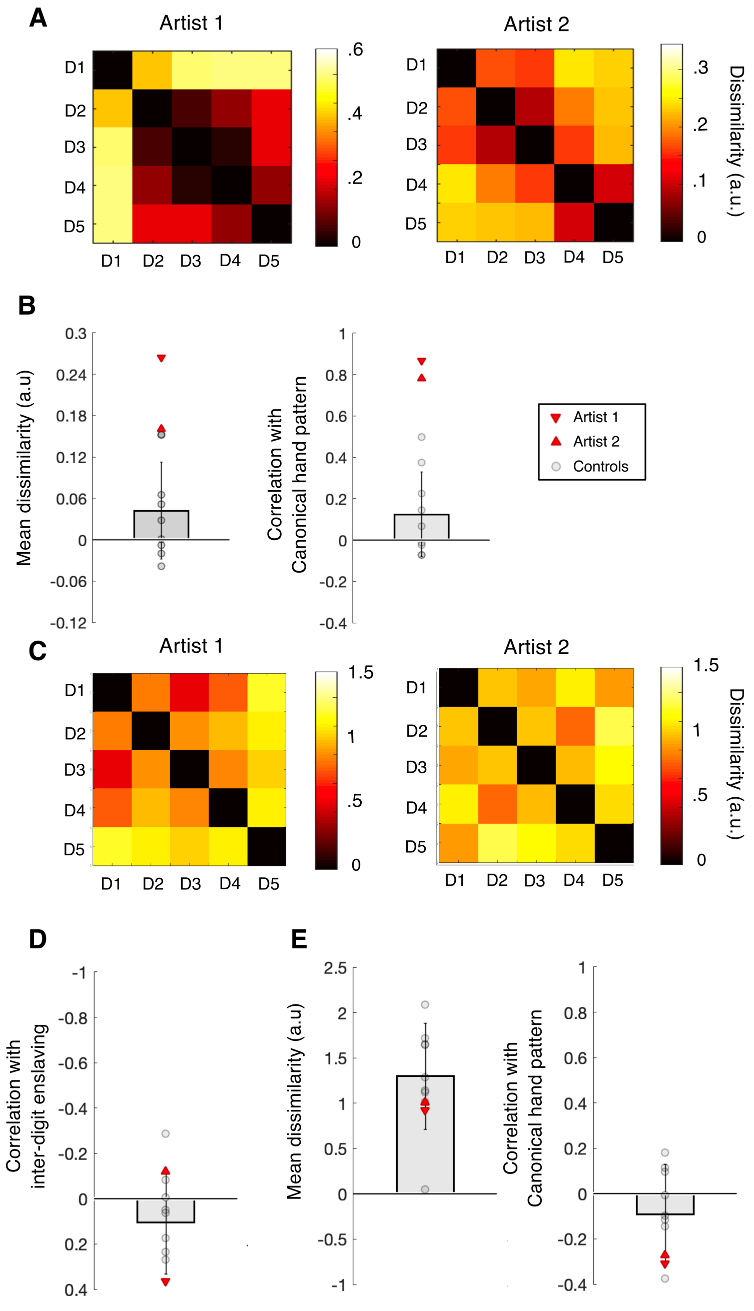

Figure S3, *Stabilising foot* representation, related to Figures 3 and 4. *Foot Area*: A) Representational dissimilarity matrices (RDM) B) RSA results. Mean dissimilarity (across the RDM) was greater for artist 1 versus controls (t(8)=2.99, p=.017), but this was not significant for artist 2 versus controls (t(8)=1.60, p=.149). Correlation with the canonical hand RDM was greater than controls for both artists (control rho, M=.125, SD=.20; artist 1 versus controls, t(8)=3.44, p=.009; artist 2 versus controls, t(8)=3.05, p=.015). *Hand Area*: C) RDMs. D) Correlation between the kinematics and the hand RDM was not different in the artists versus controls (artist 1: rho=.364, t(8)=1.08, p=.312; artist 2: rho=-.121, t(8)=-.93, p=.381). E) RSA results. Mean dissimilarity was not different for artists and controls (artist 1: t(8)=-.618, p=.554; artist 2:, t(8)=-.472, p=.650). Correlation with the canonical hand RDM was not greater than controls (artist 1: t(8)=-.929, p=.380; artist 2: t(8)=-.774, p=.461).

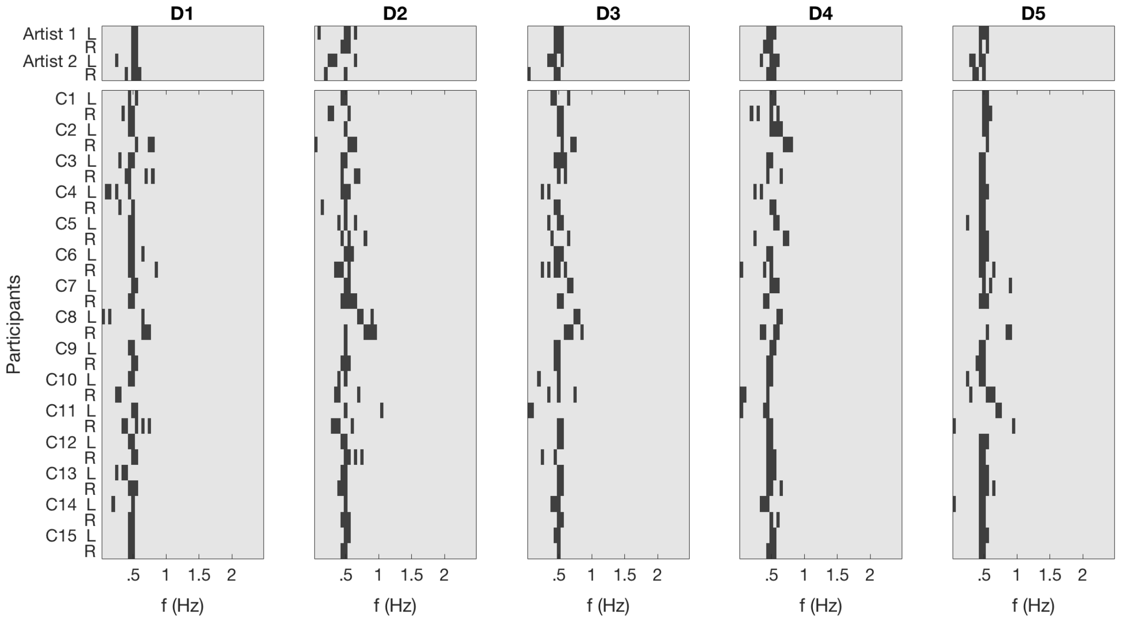

Figure S4, *Validation of kinematic task performance for the 'instructed' toe,* related to STAR Methods**.** For each participant, a Fourier transform was performed on the kinematic velocity data to isolate the frequency of movements of the toe participants were supposed to move for that block. We determined whether this frequency matched the desired frequency of 0.5Hz, i.e., one up-down cycle every 2 seconds. We found that all participants were moving at the desired frequency (showing a peak within a small frequency range around .5Hz, see above), aside from two participants who had movement frequency outside the acceptable range for one-two instructed toes – removing these participants from the analysis did not change our results. In sum, participants were performing the task as required with the instructed toe, indicating meaningful enslaving data could be gathered using this task.

Table S1. *Results for the qualitative measures used to assess the artists' complex behavioural repertoire*, related to Figure 1. (Artist 1 = ▼; Artist 2 = ▲). A) The Motor Activity Log is a clinical questionnaire previously validated to assess use of residual limbs and prostheses (e.g., Makin et al., 2013) – here adapted for feet/toes. Both artists reported using their feet/ toes to perform the majority of the 25 listed daily life tasks (very often + sometimes responses: ▼= 80%; ▲= 76%), and that this was typically very easy (not difficult responses: ▼= 80%; ▲= 66%). Please note the wording of some questions has been slightly truncated for presentation reasons. B) Tool use questionnaire assesses how often 42 different tools are used for their typical function with the upper/ lower limbs and mouth. If a tool had not been used for its typical function (i.e., only carried or transported, or not used at all), artists were asked do they think they would be able to use the tool if they had the chance. Results demonstrated both artists had used their lower limbs to use almost all tools for their typical function (▼= 88%; ▲= 90%), and that they reported they would be able to use any unused tools with their lower limbs if they had the chance. C) Adaptation of the Edinburgh Handedness Questionnaire (Oldfield, 1971) to assess preferred foot for performing 10 daily tasks (and one assessing preferred eye). Two squares for one side (left/right) indicates the artists showed full preference for use of that side, where one square for each side indicates equal preference for either the left/ right foot for performing tasks. Results indicated artist 1 was completely left foot dominant (EFI score: -100), and artist 2 was highly right foot dominant (EFI score: +92; this was sub +100 due to preferred use of left eye due to right eye damage). EFI scores range from -100 (complete left preference) to +100 (complete right preference).

A The Motor Activity Log

	Frequency			Difficulty		
Activity	Never	Some-times	Very often	Not	Med	Very
% reported	▼20% ▲24%	▼20% ▲32%	▼60% ▲44%	▼80% ▲66%	▼0% ▲10%	▼0% ▲0%
Money from wallet		▲	▼	▼▲		
Open envelope			▼▲	▼▲		
Pick up/ lift glasses			▼▲	▼▲		
Pick up/ hold up phone			▼▲	▼▲		
Wipe counter		▲	▼	▼▲		
Get out of a car			▼▲	▼▲		
Stabilize paper			▼▲	▼▲		
Carrying cup/ can	▼▲					

B Tool use

	Used for typical function			Never used for typical function	
Tool	Upper limbs	Lower limbs	Mouth	Never used	Would be able
% reported	NA	▼88% ▲90%	▼7% ▲2%	▼5% ▲7%	▼5% ▲7%
Bowl scraper		▼▲			
Calculator		▼▲			
Can opener		▼▲			
Cards		▼▲			
Chess pawn		▲		▼	▼
Comb				▼▲	▼▲
Computer mouse		▼▲			
Cooking strainer		▲	▼		

C Edinburgh footedness inventory

	Which foot used?	
Activity	Left	Right
EFI ▼ -100<br>EFI ▲ 83	▼100% ▲8%	▼0% ▲92%
Writing	▼▼	▲▲
Drawing	▼▼	▲▲
Throwing	▼▼	▲▲
Scissors	▼▼	▲▲
Toothbrush	▼▼	▲▲
Knife (alone)	▼▼	▲▲
Spoon	▼▼	▲▲
Broom (upper foot)	▼▼	▲▲

Carry bags	▼▲						Correction pen		▼▲				Striking match	▼▼	▲▲
Pull chair out		▲	▼	▼▲			Elastic band		▼▲				Opening box (lid)	▼▼	▲▲
Hold book/ turn pages		▲	▼	▼▲			Erasing gum		▼▲				Kick (preferred)	▼▼	▲▲
Keyboard/ mouse buttons			▼▲	▼▲			File		▼▲				Eye (if using one)	▼▼<br>▲▲	
Move mouse			▼▲	▼▲			Frisbee		▼▲						
Put on socks		▼▲		▼▲			Garlic Press		▼▲						
Put on shoes	▲		▼	▼			Glue stick		▼▲						
Tie shoelaces	▼▲						Hair brush		▼▲						
Put on trousers	▲		▼	▼			Hairdryer		▼▲						
Lotion/crea m on face		▼	▲	▼▲			Hand fan		▼		▲	▲			
Wash face			▼▲	▼▲			Hold punch		▼▲						
Dry face			▼▲	▼▲			Iron		▼▲						
Comb hair	▼▲						Kettle		▲	▼					
Button shirt	▼	▲		▲ (undo)	▲ (do up)		Kitchen sponge		▼▲						
Zip up coat		▼▲		▼	▲		Match		▼▲						
Peel fruit skin		▼▲		▼	▲		Nail		▼▲						
Use fork/ spoon		▼	▲	▼▲			Nail polish		▼▲						

							Paper clip		▼▲						
							Pencil sharpener		▼▲						
							Protractor		▼		▲	▲			
							Razor		▼▲						
							Rolling pin		▼▲						
							Scissors		▼▲						
							Screw		▼▲						
							Sewing needle		▼▲						
							Spinning top		▼▲						
							Stapler		▼▲						
							Syringe		▼▲						
							Tambourine		▼▲						
							Thermometer		▼	▲					
							Toaster		▲	▼					
							Tooth-brush		▼▲						
							Vegetable peeler		▼▲						
							Yo-yo		▼▲						

Table S2, *Representational similarity analysis in control regions of interest (ROIs)*, related to Figure 3. A) Controlling for Artist 2's enlarged Foot area ROI. The individually defined ROI for the Foot area (see STAR Methods) was significantly larger for both feet than controls (dextrous foot, size=1262, $t(8)=3.97$, $p=.003$, stabilising foot, size=1330, $t(8)=4.31$, $p=.002$). Artist 1's ROI was not larger (dextrous foot, size=591, $t(8)=0.58$, $p=.575$; stabilising foot, size=737, $t(8)=1.32$, $p=.219$). To confirm that this didn't impact our main findings, we repeated our analysis using an ROI of the mean controls' ROI size (dextrous: 474 mm^2, stabilising: 475 mm^2, mean control: 476±70 mm^2). Considering all of our findings were observed with the adjusted ROI size, we retained our original analysis as constraining the size was not specified a priori. B) No increased foot representation in the artists' lip area (defined using a group average activity mask for mouth movement from a different dataset (see Hamamy et al., 2017). This suggests that more 'hand-like' representation is not universal across the entire primary sensorimotor strip, but more specific to the foot and (deprived) hand area. Abbrev: A: Artist; Ctr: Control; Corr.: Correlation.

		A1 VS CTR (T)	***A1 VS CTR (P)***	***A2 VS CTR (T)***	***A2 VS CTR (P)***
**A**	Artist 2's Foot area ROI (reduced):				
	Dextrous foot				
	Mean dissimilarity	x	x	2.33	0.047
	Corr. with canonical hand	x	x	3.44	0.008
	Stabilising foot				
	Mean dissimilarity	x	x	2.33	0.047
	Corr. with canonical hand	x	x	3.44	0.008
**B**	Lip area ROI:				
	Dextrous foot				
	Mean dissimilarity	0.24	0.815	-0.09	0.93
	Corr. with canonical hand	-0.78	0.459	2.1	0.07
	Stabilising foot				
	Mean dissimilarity	-0.34	0.745	-0.4	0.701
	Corr. with canonical hand	-0.17	0.866	-0.41	0.696

Table S3, *FDR thresholds*, related to Figure 2. FDR thresholds for individual digit contrasts for the artists' dexterous foot shown in Figure 1 (Z-statistic).

	Artist 1	Artist 2
Whole-brain contrast: q(FDR)<.05	3.98	5.28
S1/M1 contrast D1 vs Others : q(FDR)<.05	3.83	3.06
D2 vs Others: q(FDR)<.05	3.43	3.68
D3 vs Others: q(FDR)<.05	2.75	3.36
D4 vs Others: q(FDR)<.05	3.41	3.17
D5 vs Others: q(FDR)<.05	3.84	3.45

Table S4, *Demographic details*, related to STAR Methods. Demographic details of participants including age, gender and dextrous foot (for the artists). Please note that not all participants performed all tests: an ‘x’ indicates participation in either the tactile, motor or fMRI testing protocols.

ID Code	Age	Gender	Dextrous foot	Tactile testing	Motor testing	fMRI testing
Artist 1	55	M	L	x	x	x
Artist 2	56	M	R	x	x	x
C1	53	F	R	x	x	x
C2	60	M	R	x	x	x
C3	48	F	R	x	x	x
C4	45	M	R	x	x	x
C5	52	F	R	x	x	x
C6	39	F	R	x	x	x
C7	75	M	R	x	x	x
C8	69	M	R	x	x	x
C9	51	F	L	x	x	x
C10	50	M	R	x	x	
C11	60	M	R	x	x	
C12	70	M	R	x	x	
C13	53	F	R	x	x	
C14	71	M	R	x	x	
C15	40	M	R	x	x	
C16	46	M	R	x		
C17	52	F	R	x		
C18	53	M	R	x		
C19	50	F	L	x		
C20	54	M	R	x		
C21	49	F	R	x		
